# Does off-pump coronary revascularization confer superior organ protection in re-operative coronary artery surgery? A meta-analysis of observational studies

**DOI:** 10.1186/1749-8090-9-115

**Published:** 2014-06-24

**Authors:** Amir H Sepehripour, Leanne Harling, Hutan Ashrafian, Roberto Casula, Thanos Athanasiou

**Affiliations:** 1 Department of Surgery and Cancer, 10th Floor QEQM Building, St Mary’s Hospital, Imperial College London, London W2 1NY, UK

**Keywords:** Re-operative, Coronary artery bypass grafting, Off-pump, Morbidity, Organ protection

## Abstract

Off-pump coronary artery bypass surgery (OPCAB) has been hypothesised to be beneficial in the high-risk patient population undergoing re-operative coronary artery bypass graft surgery (CABG). In addition, this technique has been demonstrated to provide subtle benefits in end-organ function including heart, lungs and kidney. The aims of this study were to assess whether OPCAB is associated with a lower incidence of major adverse cardiovascular and cerebrovascular events (MACCE) and other adverse outcomes in re-operative coronary surgery. Twelve studies, incorporating 3471 patients were identified by systematic literature review. These were meta-analysed using random-effects modelling. Primary endpoints were MACCE and other adverse outcomes including myocardial infarction, stroke, renal dysfunction, low cardiac output state, respiratory failure and atrial fibrillation. A significantly lower incidence of myocardial infarction, stroke, renal dysfunction, low cardiac output state, respiratory failure and atrial fibrillation was observed with OPCAB (OR 0.58; 95% CI (confidence interval) [0.39-0.87]; OR 0.37; 95% CI [0.17-0.79]; OR 0.39; 95% CI [0.24-0.63]; OR 0.14; 95% CI [0.04-0.56]; OR 0.36; 95% CI [0.24-0.54]; OR 0.41; 95% CI [0.22-0.77] respectively). Sub-group analysis using sample size, matching score and quality score was consistent with and reflected these significant findings. Off-pump coronary artery bypass grafting reduces peri-operative and short-term major adverse outcomes in patients undergoing re-operative surgery. Consequently we conclude that OPCAB provides superior organ protection and a safer outcome profile in re-operative CABG.

## Introduction

The outcome profile of off-pump coronary artery bypass surgery (OPCAB) has received much attention and analysis. Whilst recent randomised trials have popularly failed to demonstrate the beneficial effects of OPCAB in comparison to on-pump coronary artery surgery (ONCAB) with regards to mortality and major adverse cardiovascular and cerebrovascular events (MACCE), subtle benefits in end-organ function have been observed [1,2]. However, the scepticism surrounding the external validity of these trials, regarding selective patient enrolment and individual surgeon’s OPCAB experience still remains [3], and has heralded the need for closer analysis of registry data, providing a closer ‘real-life’ representation of the population [4,5].

Off-pump coronary artery bypass surgery has been demonstrated to reduce early mortality in re-operative coronary artery bypass surgery (CABG) [6]. The aim of this study is to address the question of whether OPCAB is associated with a lower incidence of MACCE and other adverse outcomes in re-operative coronary surgery.

## Review

### Materials and methods

#### ***Search***

A literature search was performed using PubMed, EMBASE and Google Scholar up to May 2013 using the MESH headings “coronary artery bypass, off-pump” and “reoperation”. Studies in English comparing outcomes in re-operative patients undergoing OPCAB versus ONCAB were included (Table 1).

**Table 1:** Pre-operative clinical characteristics for OPCAB and ONCAB groups

**Study (N)**	**N**		**Mean age**		**Previous MI**		**CHF/NYHA III/IV**		**Previous CVA/TIA**		**Hypertension**		**Diabetes**		**COPD**		**Renal impairment**		**Mean ejection fraction**		**Urgent/Emergent**	
	**Off**	**On**	**Off**	**On**	**Off**	**On**	**Off**	**On**	**Off**	**On**	**Off**	**On**	**Off**	**On**	**Off**	**On**	**Off**	**On**	**Off**	**On**	**Off**	**On**
Alamanni 2001 (123) [10]	53 (44%)	70 (56%)	66.4 (49–77)	NS	NS	NS	9 (17%)	NS	NS	NS	NS	NS	NS	NS	NS	NS	NS	NS	0.56	NS	3 (5.6%)	NS
Bergsland 1998 (289) [11]	105 (36%)	184 (64%)	66.0	65.7	76 (75.4%)	144 (78.7%)	24 (22.9%)	33 (18.1%)	12 (11.4%)	18 (9.8%)	89 (84.3%)	144 (78.7%)	25 (23.8%)	59 (32.2%)	31 (29.5%)	42 (23%)	1 (1%)	2 (1%)	0.45	0.47	8 (7.6%)	12 (6.5%)
Czerny 2003 (118) [12]	44 (37%)	74 (63%)	66.9 ± 8.9	67.1 ± 7.7	NS	NS	NS	NS	NS	NS	NS	NS	NS	NS	NS	NS	NS	NS	0.53 ± 0.14	0.57 ± 0.11	0 (0.0%)	0 (0.0%)
Dewey 2001 (432) [13]	153 (35%)	279 (65%)	64.8 ± 10.7	64.4 ± 9.78	82 (53.6%)	153 (54.8%)	19 (12.4%)	34 (12.2%)	18 (11.8%)	26 (9.3%)	92 (60.1%)	184 (66%)	37 (24.2%)	71 (25.5%)	19 (12.4%)	26 (9.3%)	2 (1.3%)	2 (0.7%)	0.48 ± 0.11	0.47 ± 0.12	0 (0.0%)	0 (0.0%)
D’Ancona 2000 (581) [14]	274 (47%)	307 (53%)	66.8 41–85)	65.5 (37–85)	NS	NS	28 (10.2%)	21 (6.8%)	27 (9.9%)	32 (10.4%)	213 (77.7%)	229 (74.6%)	60 (21.9%)	82 (26.7%)	80 (29.2%)	73 (23.8%)	10 (3.8%)	2 (0.6%)	0.47 (0.13-0.84)	0.48 (0.10-0.76)	122 (44.5%)	171 (55.7%)
Gerli 2006 (132) [15]	41 (31%)	91 (69%)	67.0 ± 8.9	65.0 ± 7.6	28 (68.3%)	51 (56%)	13 (31.7%)	21 (23.1%)	3 (7.3%)	3 (3.3%)	NS	NS	6 (14.6%)	12 (13.2%)	8 (19.5%)	13 (14.3%)	7(17.1%)	9(9.9%)	NS	NS	NS	NS
Mishra 2008 (538) [16]	332 (62%)	206 (38%)	60.4 ± 5.8	61.2 ± 6.1	153 (46.1%)	101 (49%)	23 (6.9%)	10 (4.9%)	12 (3.6%)	5 (2.4%)	159 (47.9%)	108 (52.4%)	108 (32.5%)	64 (31.1%)	25 (7.5%)	17 (8.2%)	5 (1.5%)	3 (1.4%)	0.43 ± 0.07	0.43 ± 0.07	118 (35.5%)	91 (44.2%)
Morris 2007 (771) [17]	132 (17%)	639 (83%)	67.5 ± 10.3	66.2 ± 9.4	NS	NS	37 (28%)	111 (17.4%)	34 (25.8%)	120 (18.8%)	NS	NS	52 (39.4%)	203 (31.8%)	30 (22.7%)	96 (15%)	15 (11.4%)	38 (5.9%)	0.45 ± 0.13	0.46 ± 0.12	NS	NS
Schutz 2001 (40) [18]	20 (50%)	20 (50%)	63.2 ± 9.3	67.1 ± 6.6	NS	NS	NS	NS	NS	NS	14 (70%)	12 (60%)	2 (10%)	5 (25%)	NS	NS	NS	NS	0.53 ± 0.14	0.48 ± 0.15	0 (0.0%)	0 (0.0%)
Teodori 2000 (166) [19]	54 (33%)	112 (67%)	64.7 ± 8.5	62.7 ± 8.6	NS	NS	2 (3.7%)	1 (0.9%)	1 (1.8%)	10 (8.9%)	NS	NS	NS	NS	NS	NS	NS	NS	0.54 ± 0.13	0.53 ± 0.14	20 (37%)	36 (32%)
Tugtekin 2006 (195) [20]	35 (18%)	160 (82%)	66.9 ± 7.9	66.0 ± 8.1	17 (48.6%)	96 (40%)	1 (2.9%)	11 (6.9%)	NS	NS	NS	NS	12 (34.3%)	59 (36.8%)	2 (5.7%)	10 (6.2%)	NS	NS	0.52 ± 0.14	0.55 ± 0.16	12 (34.3%)	66 (41.3%)
Vohra 2008 (86) [21]	43 (50%)	43 (50%)	65.7 ± 6.9	64.7 ± 7.7	21 (48.8%)	23 (53.5%)	12 (27.9%)	18 (41.9%)	NS	NS	27 (62.8%)	34 (79%)	10 (23.2%)	14 (32.5%)	4 (9.3%)	5 (11.6%)	1 (2.3%)	1 (2.3%)	NS	NS	11 (25.6%)	9 (20.9%)

OPCAB – off-pump coronary artery bypass; ONCAB – on-pump coronary artery bypass; MI – myocardial infarction; CHF – congestive heart failure (Ejection Fraction <40%); NYHA – New York Heart Association; CVA – cerebrovascular accident; TIA – transient ischaemic attack; COPD – chronic obstructive pulmonary disease; NS – not stated; (a) – statistical significance.

#### ***Outcomes of interest***

Primary outcomes of interest in the OPCAB and ONCAB groups were peri-operative and short-term (30-day) MACCE and other adverse outcomes including myocardial infarction, stroke, renal dysfunction, low cardiac output state, respiratory failure and atrial fibrillation.

#### ***Analysis***

Meta-analysis was performed in line with recommendations from PRISMA (Preferred Reporting Items for Systematic Reviews and Meta-Analyses) and MOOSE (Meta-Analysis of Observational Studies in Epidemiology) [7,8]. A random-effects model was used to analyse the data and the odds ratio (OR) was used as the summary statistic for binary data. Studies reporting zero events for both OPCAB and ONCAB groups were excluded from the meta-analysis.

Quantitative assessment of data validity and heterogeneity was performed using subgroup analysis for: (1) Studies with a large sample size (≥50) in each cohort, (2) Studies with a high degree of matching between OPCAB and ONCAB groups (matching score ≥ 8), and (3) Studies with a quality score ≥ 8.

#### ***Quality assessment***

The studies were assessed in two ways: (1) using a matching criteria score, and (2) using a quality assessment score based on a modified Newcastle-Ottawa scale (Table 2) [9]. The matching score was calculated for each study by attributing one point for each pre-operative characteristic for which no statistically significant difference between the OPCAB and ONCAB groups was observed (Table 3). The maximum matching score was 37 and the median score was 8. Studies with a score equal to or greater than 8 points were considered as highly-matched studies and were subgroup analysed. The modified Newcastle-Ottawa quality score was calculated for each study using the subgroup criteria of ‘selection’, ‘comparability’ and ‘outcome assessment’, and attributing stars to each study for these criteria (Table 4). The maximum quality assessment score was 15 and the median score was 8. Studies achieving a score equal to or greater than 8 points were considered as high-quality and were subgroup analysed.

**Table 2:** Quality checklist

**Checklist for quality assessment and scoring of nonrandomized studies**	
***Selection***	1. Assignment for treatment-Any criteria reported (if yes, 1 star)?
	2. How representative was the reference group (CPB) in comparison to the general population for CABG? (If yes, 1 star, no star if the patients were selected or selection of group was not described.)
	3. How representative was the treatment group (OPCAB) in comparison to the general population for CABG? (If drawn from the same community as the reference group, 1 star, no star if drawn from a different source or selection of group was not described.)
***Comparability***	4. Group comparable for 1, 2, 3, 4, 5. (If yes, 1 star was assigned for each of these characteristics. No star was assigned if the two groups differed.)
	5. Group comparable for 6, 7, 8, 9, 10. (If yes, 1 star was assigned for each of these characteristics. No star was assigned if the two groups differed.)
***Outcome assessment***	6. Clearly defined outcome of interest. (If yes, 1 star for information ascertained by record linkage or interview, no star if this information was not reported.)
	7. Follow-up (1 star if described.)
	*Comparability variables: (1) Age; (2) Gender; (3) Hypertension; (4) Diabetes; (5) Ejection fraction; (6) 3-Vessel Disease; (7) Left Main Stem Disease; (8) Urgent or Emergent Operation; (9) Viability Studies; (10) Surgeon or Hospital Volume*

**Table 3:** Study matching score

**Study**	**Inclusion criteria**		**Exclusion criteria**		**Matching criteria**	**Matching score (Max 37)**
	**Off**	**On**	**Off**	**On**		
Alamanni [10]	A, F	A	NS	NS	NS	0
Bergsland [11]	A, K	A, K	NS	NS	8, 13, 15, 16, 18, 20, 21,25, 28, 29, 31	11
Czerny [12]	A, B, K	A, B, K	D, G	D, G	2, 8, 10, 26, 32, 33	6
Dewey [13]	A, F, G	A	E, F	A, B, E, F	2, 5, 8, 13, 14, 15, 16, 18, 20, 21, 22	11
D’Ancona [14]	A	A	NS	NS	1, 2, 3, 5, 8, 12, 15, 16, 18, 19, 20, 21, 24, 25, 28, 29	16
Gerli [15]	A, B	A, B	NS	NS	2, 3, 9, 13, 16, 18, 20, 21	8
Mishra [16]	A, I, K	A, I, K	D, E, I, J	J	NS	0
Morris [17]	A, K	A, K	NS	NS	2, 3, 5, 6, 7, 8, 10, 13, 15, 16, 18, 19, 20, 21, 31, 37	16
Schutz [18]	A, I	A, K	D, B	D, B	2, 3, 8, 15, 16, 17, 18	7
Teodori [19]	A	A	NS	NS	2, 3, 8, 21, 22, 26, 28, 31	8
Tugtekin [20]	A, K	A, K	J	J	2, 3, 8, 9, 10, 11, 13, 16, 20, 22, 24, 25, 26	13
Vohra [21]	A, B, C	A, B, C	D, F	D, F	3, 6, 7, 9, 10, 12, 13, 15, 16, 17, 18, 20, 22, 24, 29, 30, 31, 32	18

Inclusion Criteria: (A) Re-do CABG, (B) Elective operation; (C) Previous CPB CABG; (D) Single-vessel disease; (E) Multi-vessel disease; (F) High-risk for CPB; (G) Significant medical co-morbidities; (H) Age >75; (I) Symptomatic CAD not managed by medical therapy; (J) Angiographically non-calcified non-intramyocardial distal LAD and patent LIMA; (K) Discretion of the surgeon.

Exclusion Criteria: (A) High-risk for CPB; (B) Significant medical co-morbidities; (C) Religious convictions that precluded blood transfusion; (D) Emergency operation; (E) Haemodynamic instability; (F) Cardiogenic shock; (G) Unstable angina; (H) Age <75; (I) Poor quality of distal coronary target vessels; (J) Discretion of the surgeon.

Matching Criteria: (1) Number of patients; (2) Age; (3) Gender; (4) Body Mass Index; (5) Congestive Heart Failure; (6) CCS Class III/IV; (7) NYHA Class III/IV; (8) Ejection Fraction; (9) Ejection Fraction <30%; (10) Number of Diseased Vessels; (11) 3-Vessel Disease; (12) Left Main Stem Disease; (13) Previous MI; (14) Previous Valve Repair/Replacement; (15) Hypertension; (16) Diabetes; (17) Hypercholestrolaemia; (18) Renal Impairment/Failure; (19) Pre-Op Dialysis; (20) COPD; (21) Cerebrovascular Disease; (22) Peripheral Vascular Disease; (23) Elective Operation; (24) Urgent Operation; (25) Emergency Operation; (26) Time from Previous CABG; (27) Frequency of Patent, Significantly Stenosed (>70%) or Occluded Previous Grafts; (28) Calcified Ascending Aorta; (29) Pre-Op IV GTN; (30) Pre-Op IV Inotropes; (31) Pre-Op IABP; (32) EuroSCORE; (33) Parsonnet Score; (34) Cleveland Clinic Score; (35) Viability Studies; (36) Hospital Volume; (37) Surgeon Volume.

**Table 4:** Study quality score

**Study**	**Quality assessment – selection**			**Quality assessment – comparability**		**Quality assessment – outcome assessment**		**Quality assessment - score (out of 15)**
	**1**	**2**	**3**	**4**	**5**	**6**	**7**	
Alamanni [10]	*	*	*	-	-	*	-	4
Bergsland [11]	*	*	*	***	*	*	-	8
Czerny [12]	*	*	*	**	-	*	*	7
Dewey [13]	*	*	*	****	-	*	-	8
D’Ancona [14]	*	*	*	*****	**	*	-	11
Gerli [15]	*	*	*	****	-	*	-	8
Mishra [16]	*	*	*	-	-	*	-	4
Morris [17]	*	*	*	*****	*	*	-	10
Schutz [18]	*	*	*	*****	-	*	*	10
Teodori [19]	*	*	*	***	-	*	*	8
Tugtekin [20]	*	*	*	****	**	*	*	11
Vohra [21]	*	*	*	****	**	*	*	11

*: the number of points each study is given in each of the categories.

## Results

**Figure 1:**
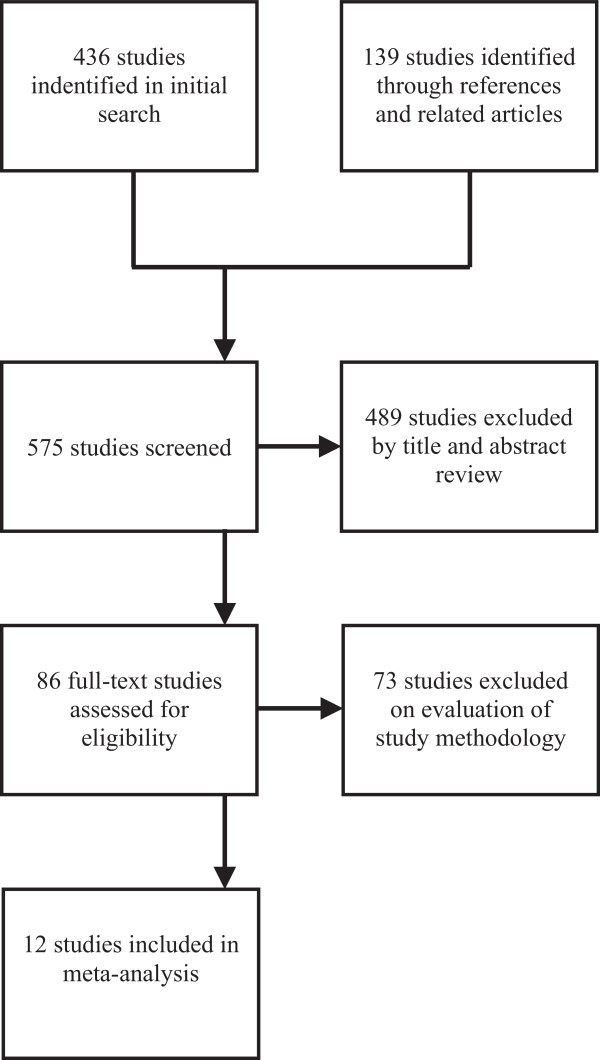
Search strategy.

Twelve studies were identified by systematic search to fulfil the inclusion criteria (Figure 1) [10-21]. These studies included 3,471 patients, 1,286 of whom underwent OPCAB and 2,185 underwent ONCAB. All studies used standard cardiopulmonary bypass (CPB) circuits without the use of minimised systems. There were several variations in myocardial arrest and cardioplegia strategy within the ONCAB group. Of the 12 studies, five used cold blood cardioplegia [12,14-16,21], one used cold crystalloid [10], 2 used a combination of cold blood and cold crystalloid [11,20] and one study used a combination of cold blood, warm blood and hypothermic ventricular fibrillation [19]. The majority of studies (5/9) administered a combination of antegrade and retrograde cardioplegia [11,14,16,17,21]. Two studies used only an antegrade approach [19,20] and the remainder did not specify their technique.

### Primary outcomes

#### ***Myocardial infarction***

**Figure 2:**
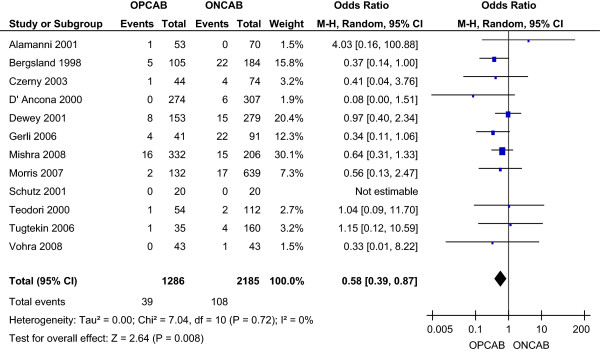
Myocardial infarction for OPCAB and ONCAB groups.

The incidence of acute myocardial infarction was 3.03% in the OPCAB group and 4.94% in the ONCAB group, as reported by all studies. This reduction with OPCAB was statistically significant using a random effects model (OR 0.58; 95% CI (confidence interval) 0.39-0.87) (Figure 2). No significant heterogeneity was found between the studies.

#### ***Stroke***

**Figure 3:**
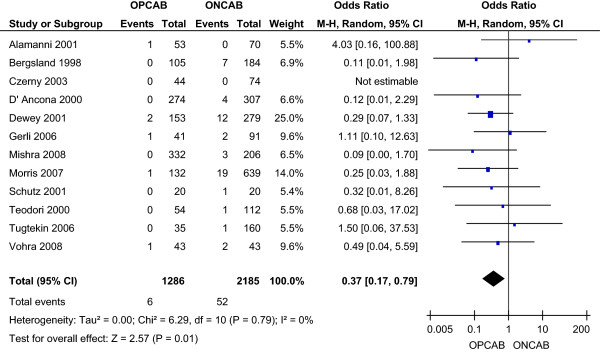
Stroke for OPCAB and ONCAB groups.

The incidence of stroke was 0.47% in the OPCAB group and 2.38% in the ONCAB group, as reported by all studies. This reduction with OPCAB was statistically significant using the random effects model (OR 0.37; 95% CI 0.17-0.79) (Figure 3), without significant heterogeneity between the studies.

#### ***Renal failure***

**Figure 4:**
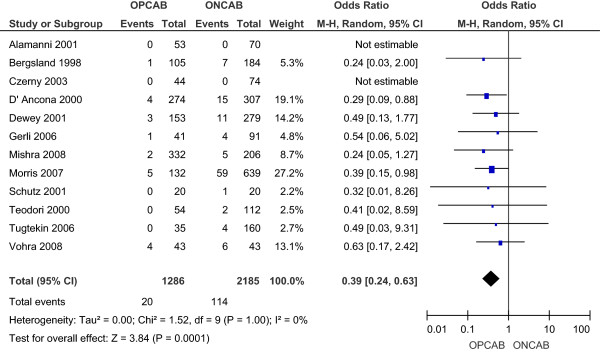
Renal failure for OPCAB and ONCAB groups.

The incidence of acute renal failure with or without the need for renal replacement therapy was 1.56% in the OPCAB group and 5.22% in the ONCAB group, as reported by ten studies [11,13-21]. This reduction with OPCAB was statistically significant using the random effects model (OR 0.39; 95% CI 0.24-0.63) (Figure 4), without significant heterogeneity between the studies.

#### ***Low cardiac output state***

**Figure 5:**
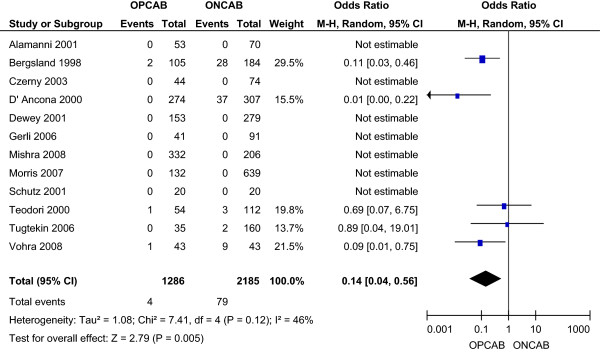
Low cardiac output state for OPCAB and ONCAB groups.

The incidence of the use of peri- or post-operative intra-aortic balloon pump (IABP) secondary to a low cardiac output state was 0.31% in the OPCAB group and 3.62% in the ONCAB group, as reported by five studies [11,14,19-21]. This reduction with OPCAB was statistically significant using the random effects model (OR 0.14; 95% CI 0.04-0.56) (Figure 5), without significant heterogeneity between the studies.

#### ***Respiratory failure***

**Figure 6:**
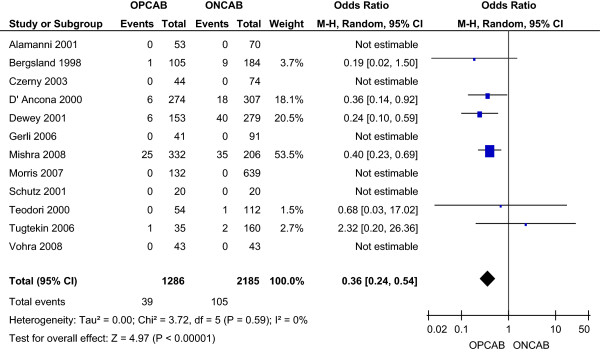
Respiratory failure for OPCAB and ONCAB groups.

The incidence of acute respiratory failure or acute respiratory distress syndrome (ARDS) was 3.03% in the OPCAB group and 4.81% in the ONCAB group, as reported by six studies [11,13,14,16,19,20]. This reduction with OPCAB was statistically significant using the random effects model (OR 0.36; 95% CI 0.24-0.54) (Figure 6), without significant heterogeneity between the studies.

#### ***A trial fibrillation***

**Figure 7:**
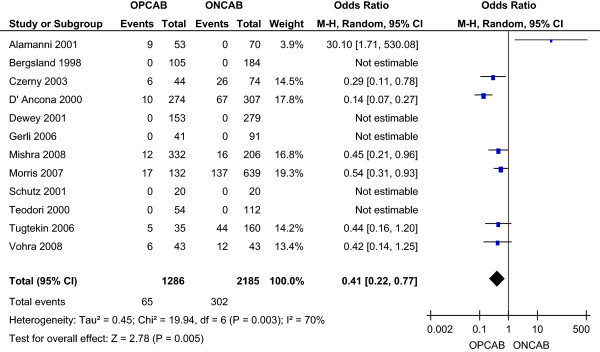
Atrial fibrillation for OPCAB and ONCAB groups.

The incidence of post-operative atrial fibrillation was 5.05% in the OPCAB group and 13.8% in the ONCAB group, as reported by seven studies [10,12,14,16,17,20,21]. This reduction with OPCAB was statistically significant using the random effects model (OR 0.41; 95% CI 0.22-0.77) (Figure 7), without significant heterogeneity between the studies.

### Subgroup analysis

#### ***Sample size***

Seven studies had a large sample size (≥50) in each cohort and were in this subgroup analysis [10,11,13,14,16,17,19].

Myocardial infarction was again significantly lower in the OPCAB compared to the ONCAB group (2.99% vs. 4.28%; OR 0.63; 95% CI 0.41-0.99). Incidence of stroke was again significantly lower with OPCAB (0.36% vs. 2.56% ONCAB; OR 0.28; 95% CI 0.11-0.69). Similarly, the incidence of renal failure was again significantly lower with OPCAB (1.36% vs. 5.51% ONCAB; OR 0.35; 95% CI 0.20-0.60). Four studies [11,14,16,19] reported a significantly reduced incidence of low cardiac output states with OPCAB (0.27% vs. 3.78% ONCAB; OR 0.11; 95% CI 0.01-0.87). Five studies [11,13,14,16,19] reported a significantly reduced incidence of respiratory failure/ARDS with OPCAB (3.45% vs. 5.73% ONCAB; OR 0.35; 95% CI 0.23-0.52). Four studies [10,14,16,17] reported a reduced incidence of atrial fibrillation with OPCAB (4.35% vs. 12.24% ONCAB; OR 0.51; 95% CI 0.17-1.51).

#### ***Matching score and quality score***

Eight studies were attributed a matching score of 8 or greater and were included in this subgroup analysis [11,13-15,17,19-21]. Nine studies were attributed a quality score of 8 or greater and were included in this analysis [11,13-15,17-21]. All studies with high matching score were also found to have high quality score and therefore subgroup analysis for matching and quality scores are considered together.

Myocardial infarction was again significantly lower in the OPCAB compared to the ONCAB group (2.51% vs. 4.9%; OR 0.54; 95% CI 0.33-0.89). Incidence of stroke was again significantly lower with OPCAB (0.6% vs. 2.64% ONCAB; OR 0.36; 95% CI 0.16-0.82). Similarly, the incidence of renal failure was again significantly lower with OPCAB (2.15% vs. 5.95% ONCAB; OR 0.40; 95% CI 0.24-0.68). Five studies [11,14,19-21] reported a significantly reduced incidence of low cardiac output states with OPCAB (0.48% vs. 4.35% ONCAB; OR 0.14; 95% CI 0.04-0.56). Five studies [11,13,14,19,20] reported a significantly reduced incidence of respiratory failure/ARDS with OPCAB (1.67% vs. 3.86% ONCAB; OR 0.33; 95% CI 0.18-0.59). Four studies [14,17,20,21] reported a significantly reduced incidence of atrial fibrillation with OPCAB (7.85% vs. 22.6% ONCAB; OR 0.33; 95% CI 0.16-0.70).

### Comment

This meta-analysis demonstrates that OPCAB reduces the incidence of major adverse events in the peri-operative and short-term follow-up period. Significant reductions were observed in the rates of myocardial infarction, stroke, renal failure, low cardiac output state, respiratory failure and atrial fibrillation. These observed beneficial effects of OPCAB remained consistent in the subgroup analysis of large sample size, high matching score and high quality score studies.

Re-operative coronary artery surgery poses a great risk to an already high-risk, older patient cohort with potentially higher co-morbidity and end-organ disease. The challenging technical aspects of the operation itself; including sternal re-entry, epicardial adhesions, patent or diseased in-situ grafts, more advanced coronary and aortic disease burden and the sufficiency of myocardial protection are all the challenging technical aspects contributing to the high-risk nature of the operation. These complexities of re-operative coronary surgery have inevitably not permitted the inclusion of these patients in randomised trials.

A very clear spectrum of difficulty is observed in different patients undergoing re-operative coronary artery surgery, providing a spectrum of challenges. Baseline co-morbidities, ventricular function and the quality of target coronary vessels are some of the factors affecting the decision to undertake OPCAB, the risk of conversion to ONCAB, the degree of target vessel revascularisation, the effect on target organ preservation and ultimately the outcome profile [6].

The conduct of OPCAB, whilst in itself very challenging, prompting scepticism regarding surgeon experience and expertise, does provide very clear protection for the heart, the brain and other organs. Firstly, avoiding manipulation of the aorta and previous bypass grafts in OPCAB reduces the risk of coronary and cerebral embolisation. Secondly, providing sufficient myocardial protection in the setting of extensive coronary disease is challenging and often requires a retrograde approach. Utilisation of intra-coronary shunts in OPCAB reduce the ischaemic time and consequent myocardial stunning. Thirdly, the avoidance of extra-corporeal membrane circulation eliminates the systemic inflammatory response observed with CPB, protecting predominantly the lungs and the kidneys as well as all other organs. Finally, the elimination of the CPB-induced coagulopathy may decrease the need for transfusion and potentially reduce re-exploration for bleeding [6]. However, it is crucial to consider that, given the spectrum of difficulty observed with re-operative coronary surgery and the factors affecting the decision to undertake OPCAB, appropriate patient selection is of paramount importance, whereby OPCAB is not necessarily the most appropriate technique for all patients.

In a recent analysis of the Society of Thoracic Surgeons Adult Cardiac Surgery Database by Ghanta et al. [22], outcomes of 72,431 patients undergoing isolated reoperative coronary artery bypass grafting were observed over the period 2000 to 2009. Risk-adjusted rates of stroke decreased from 1.9% in 2000 to 1.6% in 2009 (relative risk reduction -16.5%, *p* < 0.001); of renal failure decreased from 5.5% to 4.9% (-15.4%, *p* < 0.004); of prolonged ventilation increased from 11.6% to 15.3% (31.4%, *p* = 0.532); and of atrial fibrillation increased from 19.0% to 19.9% (4.8%, *p* = 0.532); amongst other outcome measures of morbidity observed. The OPCAB outcome measures of our study compare favourably with those of Ghanta and colleagues, the largest report to date analysing isolated reoperative CABG.

OPCAB has been demonstrated to reduce early mortality in re-operative CABG [6]. The aim of this study was to explore beyond these effects on mortality and attempt to ascertain whether OPCAB can reduce MACCE and other adverse outcomes in re-operative CABG by means of providing more superior organ protection. All of the studied outcome measures were demonstrated to be significantly reduced with OPCAB, emphasising the superior organ protection provided by the technique.

#### ***Limitations***

The issue of surgeon experience and expertise with OPCAB surgery has been a source of much debate and criticism [2]. This challenging technique has a very pronounced learning curve and requires time and experience in order for the outcomes to be comparable. The same concept applies for re-operative surgery. Whilst not under the same degree of scepticism, owing to the lack of randomised trials, re-operation itself is an even bigger challenge, requiring particular experience and is more time consuming. In order to improve the validity and reliability of the results of this study, surgeon experience in both OPCAB and re-operative CABG will need to be accounted and adjusted for.

A further limitation of this study is the short follow-up period of analysis. We have demonstrated significant reduction in 30-day adverse outcomes using OPCAB, however in order to allow generalisation of these results regarding the outcome profile of OPCAB in re-operative CABG, mid- and long-term analysis of adverse outcomes are required.

A further limitation of this study which is required to be taken into account is that OPCAB is not necessarily the most appropriate technique for all patients undergoing re-operative CABG. This technique can only be demonstrated to be superior in the setting of suitable coronary target vessels, a low risk of conversion to ONCAB and minimal requirement for manipulation of the heart, allowing for target vessel revascularisation.

## Conclusions

Off-pump coronary artery bypass grafting reduces peri-operative and short-term major adverse outcomes in patients undergoing re-operative coronary artery surgery. These outcomes include myocardial infarction, stroke, renal failure, low cardiac output state, respiratory failure and new-onset atrial fibrillation. Consequently we conclude that OPCAB provides superior organ protection and a safer outcome profile in re-operative CABG.

## Abbreviations

ARDS: Acute respiratory distress syndrome; CABG: Coronary artery bypass graft; CI: Confidence interval; CPB: Cardiopulmonary bypass; IABP: Intra-aortic balloon pump; MACCE: Major adverse cardiovascular and cerebrovascular events; MOOSE: Meta-analysis of observational studies in epidemiology; ONCAB: On-pump coronary artery bypass; OPCAB: Off-pump coronary artery bypass; OR: Odds ratio; PRISMA: Preferred reporting items for systematic reviews and meta-analyses.

## Competing interests

The authors declare that they have no competing interests.

## Authors’ contributions

AS - made substantial contributions to conception and design, acquisition of data, analysis and interpretation of data; involved in drafting the manuscript and revising it critically for important intellectual content; given final approval of the version to be published; and agree to be accountable for all aspects of the work in ensuring that questions related to the accuracy or integrity of any part of the work are appropriately investigated and resolved. LH - made substantial contributions to conception and design, acquisition of data, analysis and interpretation of data; involved in drafting the manuscript and revising it critically for important intellectual content; given final approval of the version to be published; and agree to be accountable for all aspects of the work in ensuring that questions related to the accuracy or integrity of any part of the work are appropriately investigated and resolved. HA - made substantial contributions to conception and design; involved in drafting the manuscript and revising it critically for important intellectual content; and agree to be accountable for all aspects of the work in ensuring that questions related to the accuracy or integrity of any part of the work are appropriately investigated and resolved. RC - made substantial contributions to conception and design; involved in drafting the manuscript and revising it critically for important intellectual content; and agree to be accountable for all aspects of the work in ensuring that questions related to the accuracy or integrity of any part of the work are appropriately investigated and resolved. TA - made substantial contributions to conception and design; involved in drafting the manuscript and revising it critically for important intellectual content; given final approval of the version to be published; and agree to be accountable for all aspects of the work in ensuring that questions related to the accuracy or integrity of any part of the work are appropriately investigated and resolved. All authors read and approved the final manuscript.
